# Fulminant Hepatic Failure With Minimal Alcohol Consumption in a 25-Year-Old Female With Hereditary Hemochromatosis: A Rare Case

**DOI:** 10.7759/cureus.44544

**Published:** 2023-09-01

**Authors:** Ali Tariq Alvi, Luis E Santiago, Zahid Nadeem, Ali Chaudhry

**Affiliations:** 1 Internal Medicine, HCA Florida Northwest Hospital, Margate, USA; 2 Critical Care, HCA Florida Northwest Hospital, Margate, USA

**Keywords:** young patients, iron overload, alcohol use, fulminant hepatic failure, hereditary hemochromatosis

## Abstract

Hereditary hemochromatosis (HH) is an inherited disorder in which organ damage and other clinical manifestations are commonly seen in patients with a homozygous mutation involving *C282Y* of the *HFE* gene, causing increased iron absorption in the intestine. The liver is the primary site of iron deposition, and excessive iron overload can eventually lead to hepatic cirrhosis. Patients who drink significant amounts of alcohol are more likely to develop cirrhosis, and in females, it is commonly seen after menopause. We describe a young female with hereditary hemochromatosis who developed fulminant hepatic failure with minimal alcohol consumption at age 25.

## Introduction

Hereditary hemochromatosis (HH) is an autosomal recessive disorder primarily associated with a mutation in *C282Y* of the *HFE* gene (chromosome 6p21.3). The other less common mutations involve *H63D* and *S65C* in the *HFE* gene. The homozygosity of *C282Y* occurs in 0.3%-0.6% of European people and is responsible for hemochromatosis-related iron overloading in 90% of Caucasians [1,2]. Patients with symptoms or physical findings suggestive of disease, or positive family history should undergo serum testing of ferritin and transferrin levels. If these levels are elevated, *HFE* mutation testing is performed as a confirmatory test [3]. Cirrhosis or hepatocellular cancer is not uncommon in older males with hereditary hemochromatosis, with one to 10 males likely to develop severe liver disease during their lifetime unless the disease is detected early and treatment is initiated promptly. Liver disease can be prevented by periodic phlebotomy to reach target serum ferritin levels and by monitoring annual serum ferritin [4]. Multiple studies have explained the association between alcohol use and cirrhosis in patients with hereditary hemochromatosis. Most of them highlight the fact that alcohol consumption does increase the risk of cirrhosis in such patients [5,6]. However, we could not find any study that approximates the average length of time before cirrhosis onset in such patients and how this variable is associated with young age.

## Case presentation

**Figure 1:**
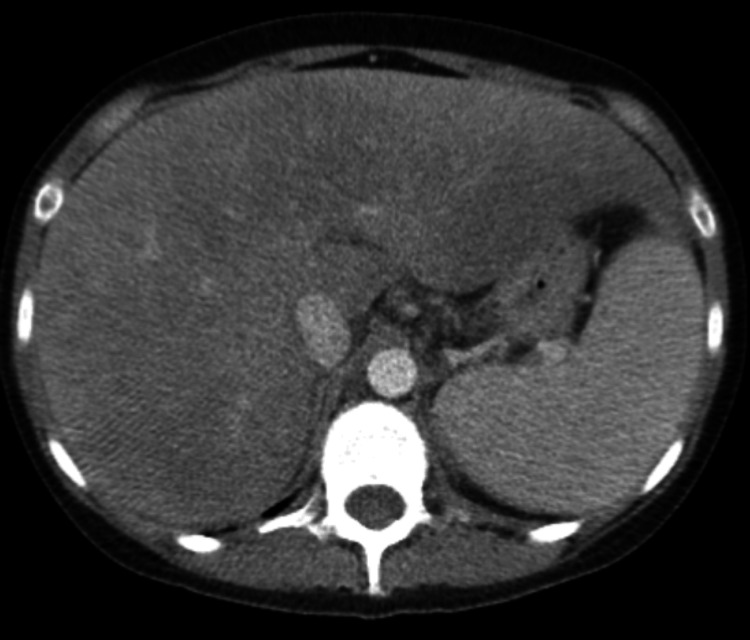
Axial view of computed tomography (CT) of abdomen showing hepatomegaly and splenomegaly.

**Figure 2:**
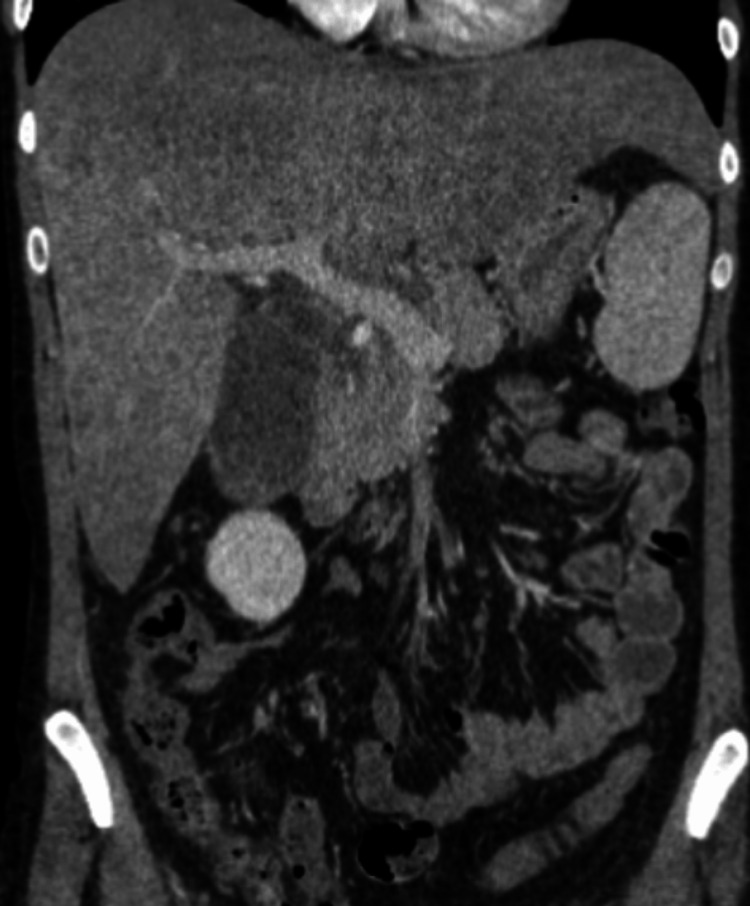
Coronal view of computed tomography (CT) of abdomen showing massive hepatomegaly

We describe a 25-year-old female with a past history of hereditary hemochromatosis who presented in the emergency department with a one-week history of hematemesis and yellowing of the skin. She also had associated abdominal pain with no diarrhea or nausea. She reported consuming one glass of wine a week for the last year. She had a family history of hereditary hemochromatosis in her father, and she was diagnosed herself many years ago with positive *HFE* genetic testing. Initially, she used to get phlebotomy every couple of weeks, but she started getting it less frequently over the last two years. She reported having regular menstrual cycles. On review of her home medications, she was only taking fluoxetine and multivitamins. She denied using herbal medicines or other illicit drugs. In the ER, she was afebrile, with a heart rate of 56 beats per minute, blood pressure of 101/57 mmHg, and oxygen saturation of 97% on room air. Her laboratory investigations revealed a white blood cell (WBC) count of 20,500/mm^3^, hemoglobin of 6.1 g/dl, platelet count of 93000, sodium of 113 mmol/L, potassium of 3.6 mmol/L, chloride of 80 mmol/L, bicarbonate of 11 mmol/L, blood urea nitrogen of 50 mg/dl, creatinine of 7.4 mg/dl, calcium of 7.3 mg/dl, phosphorous of 7.6 mg/dl, magnesium of 1.3 mg/dl, total bilirubin of 35.7 mg/dl, conjugated bilirubin of 26.2 mg/dl, aspartate aminotransferase (AST) of 166 units/L, alanine aminotransferase (ALT) of 39 units/L, alkaline phosphatase of 120 units/L, ammonia of 30 micromol/L, albumin of 3.4 g/dl, ferritin of 2,850 ng/ml. Her coagulation profile showed prothrombin time (PT) of 28.9 seconds, international normalised ratio (INR) of 2.5, activated partial thromboplastin time (aPTT) of 50 seconds, and fibrinogen of 173 mg/dl. Her pregnancy test was negative. The other tests to evaluate hepatic failure, including viral hepatitis serologies, acetaminophen levels, anti-nuclear antibody (ANA) screen, anti-smooth muscle antibody, anti-mitochondrial antibody, serum ceruloplasmin, and urine drug screen, were unremarkable. Computed tomography (CT) scan of the abdomen showed hepatomegaly with hepatic steatosis, splenomegaly, esophageal varices, and gallbladder distension (Figures 1, 2). 

**Figure 3:**
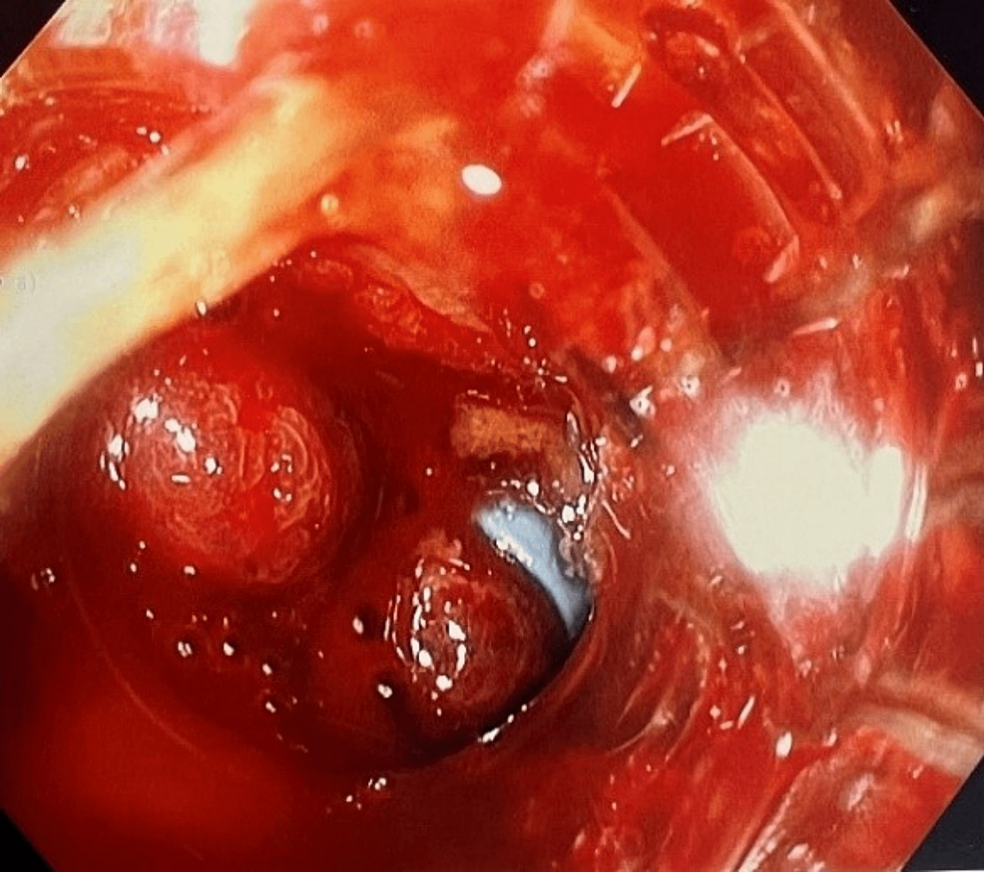
Endoscopy (EGD) showing profuse bleeding with band placement

The physical examination was significant for scleral icterus, jaundice, distended abdomen, dry blood around teeth, and hepatomegaly. Due to the poor clinical status, she was admitted to the intensive care unit (ICU) for close monitoring, where she was intubated on the first night prior to the endoscopy. The endoscopy showed large esophageal varices with active bleeding in the distal esophagus, which required the placement of seven bands (Figure 3).

The bleeding could not be stopped, and she continued to bleed for the next few days. She received 10 units of packed red blood cells, five units of fresh frozen plasma (FFP), one unit of platelets, and one unit of cryoprecipitate. She stayed in the ICU for five days and was eventually transferred to the nearest transplant center for liver transplantation.

## Discussion

In patients with hereditary hemochromatosis, there is a 24-fold increased rate of iron accumulation in men compared to women. Among those, young patients rarely present with significant iron overload symptoms. Females commonly present after menopause due to less iron overload during premenopausal years, and males generally present after 40 years of age. Clinical manifestations and end-organ damage occur in only 10% of patients homozygous for *C282Y*. Symptoms are typically absent in the early stages but, if present, include arthralgias, weakness, and lethargy. Manifestations later in life include cirrhosis, hepatocellular cancer, hypogonadism, cardiomyopathy, dysrhythmia, and diabetes mellitus [3].

Excess iron in the body produces reactive oxygen species that exceed the normal antioxidant defenses, leading to lipid membrane peroxidation, causing liver cell damage [7]. As the liver attempts to repair this injury, the resulting cellular response leads to the activation of fibro-genic pathways [8]. The progression from fibrosis to cirrhosis is crucial in managing these patients, as cirrhosis causes a significant reduction in life expectancy, as the five-year survival rate is close to 50% in unmanaged cirrhotic patients [5,9]. Also, combining two or more toxic insults can accelerate the process, as seen with alcohol consumption, which significantly increases the development of cirrhosis in patients with iron overload [5,6]. According to one study, the amount of iron accumulation in the liver associated with the development of cirrhosis is almost 233-675 micromoles per gram, and patients with hemochromatosis who drink more than 60 grams of alcohol per day are nine times more likely to develop cirrhosis than those patients who drink less than that amount [6]. Even though the above studies indicate an association of alcohol in the development of cirrhosis in hereditary hemochromatosis, there is no data that could explain the number of years it will take to develop. Also, we did not come across any other case of fulminant hepatic failure developing in a patient with hereditary hemochromatosis in this age group.

## Conclusions

Cirrhosis or late-stage hepatic failure is usually seen after 40 years of age in males and after menopause in females with underlying hereditary hemochromatosis. Alcohol consumption does increase the risk of cirrhosis in such patients, but it is unlikely to see cirrhosis with fulminant hepatic failure in younger patients. The patient with underlying hereditary hemochromatosis described in this case developed life-threatening fulminant hepatic failure at age 25, with minimal alcohol consumption, which has not been reported in the literature before. This case report will hopefully drive more research on the association between alcohol and the pathophysiology of the development of cirrhosis in young patients with hereditary hemochromatosis.
